# Cardiovascular magnetic resonance demonstration of the spectrum of morphological phenotypes and patterns of myocardial scarring in Anderson-Fabry disease

**DOI:** 10.1186/s12968-016-0233-6

**Published:** 2016-03-31

**Authors:** Djeven Parameshvara Deva, Kate Hanneman, Qin Li, Ming Yen Ng, Syed Wasim, Chantal Morel, Robert M. Iwanochko, Paaladinesh Thavendiranathan, Andrew Michael Crean

**Affiliations:** Department of Medical Imaging, St. Michael’s Hospital, University of Toronto, 30, Bond Street, Toronto, ON M5B 1W8 Canada; Department of Medical Imaging, Peter Munk Cardiac Centre, Toronto General Hospital, University of Toronto, 585 University Ave, Toronto, ON M5G 2N2 Canada; Division of Cardiology, Peter Munk Cardiac Centre, Toronto General Hospital, University of Toronto, 585 University Ave, Toronto, ON M5G 2N2 Canada; Fred A. Litwin Centre in Genetic Medicine, University Health Network & Mount Sinai Hospital, 60 Murray St., 3rd floor, Room 400, Toronto, M5T 3L9 ON Canada; Department of Diagnostic Radiology, The University of Hong Kong, Queen Mary Hospital, 102, Pokfulam Road, Hong Kong; The Hospital for Sick Children, 555, University Avenue, Toronto, ON M5G 1X8 Canada; Division of Cardiology, Toronto Western Hospital, 399 Bathurst St, Toronto, ON M5T 2S8 Canada

**Keywords:** Cardiomyopathy, Hypertrophy, Cardiovascular magnetic resonance, Anderson-Fabry disease, Left ventricular morphology, Myocardial scar, Late gadolinium enhancement

## Abstract

**Background:**

Although it is known that Anderson-Fabry Disease (AFD) can mimic the morphologic manifestations of hypertrophic cardiomyopathy (HCM) on echocardiography, there is a lack of cardiovascular magnetic resonance (CMR) literature on this. There is limited information in the published literature on the distribution of myocardial fibrosis in patients with AFD, with scar reported principally in the basal inferolateral midwall.

**Methods:**

All patients with confirmed AFD undergoing CMR at our center were included. Left ventricular (LV) volumes, wall thicknesses and scar were analyzed offline. Patients were categorized into 4 groups: 1) no wall thickening; 2) concentric hypertrophy; 3) asymmetric septal hypertrophy (ASH); and 4) apical hypertrophy. Charts were reviewed for clinical information.

**Results:**

Thirty-nine patients were included (20 males [51 %], median age 45.2 years [range 22.3–64.4]). Almost half (17/39) had concentric wall thickening. Almost half (17/39) had pathologic LV scar; three quarters of these (13/17) had typical inferolateral midwall scar. A quarter (9/39) had both concentric wall thickening and typical inferolateral scar. A subgroup with ASH and apical hypertrophy (*n* = 5) had greater maximum wall thickness, total LV scar, apical scar and mid-ventricular scar than those with concentric hypertrophy (*n* = 17, *p* < 0.05). Patients with elevated LVMI had more overall arrhythmia (*p* = 0.007) more ventricular arrhythmia (*p* = 0.007) and sustained ventricular tachycardia (*p* = 0.008).

**Conclusions:**

Concentric thickening and inferolateral mid-myocardial scar are the most common manifestations of AFD, but the spectrum includes cases morphologically identical to apical and ASH subtypes of HCM and these have more apical and mid-ventricular LV scar. Significant LVH is associated with ventricular arrhythmia.

**Electronic supplementary material:**

The online version of this article (doi:10.1186/s12968-016-0233-6) contains supplementary material, which is available to authorized users.

## Background

X-linked mutations in the α-galactosidase gene cause Anderson Fabry disease (AFD), a lysosomal storage disorder [1]. Although genetic testing is used to diagnose AFD, cardiovascular magnetic resonance (CMR) is often performed for accurate volumetric and functional analysis in this disease and to characterize the myocardium. It is recognized from the echocardiography literature that AFD may mimic the morphological characteristics of the various subtypes of hypertrophic cardiomyopathy. In addition, a small proportion of AFD patients have no extracardiac manifestations and therefore, they may be misdiagnosed as having hypertrophic cardiomyopathy (HCM) or hypertensive heart disease [2–8]. Although it is known that AFD can mimic the morphologic manifestations of HCM on echocardiography [5–7, 9–13], CMR literature on this subject is lacking. There is limited information in the published literature on the distribution of myocardial late gadolinium enhancement (LGE) in patients with AFD, with enhancement reported principally in the basal inferolateral midwall [14–16]. We aimed to use the superior myocardial characterization and spatial resolution of CMR to catalogue the full spectrum of LGE patterns and distribution of left ventricular wall thickening seen in this rare disease.

## Methods

### Patient population

Institutional research and ethics board approval was obtained for this retrospective study (REB#: 12-5646-AE) from the University Health Network Research and Ethics Board, and the requirement to obtain individual patient consent was waived. Patients with confirmed AFD, identified through our metabolic genetic disease clinic between 1/1/2000 and 31/12/2013, who had CMR (with LGE sequences) at our institution, were included in this study. We included only patients with disease-causing AFD mutation or positive leukocyte alpha galactosidase A activity test confirming a diagnosis of AFD. Exclusion criteria were age <18 at CMR acquisition, more than mild aortic stenosis, disease-causing HCM mutations and LGE due to myocardial infarction. Arrhythmia was defined as documented ventricular arrhythmia or atrial fibrillation. Ventricular arrhythmia was defined as documented non-sustained ventricular tachycardia (>6 beats at a minimum of 120 beats/min) and/or an episode (or episodes) of sustained ventricular tachycardia (ventricular tachycardia lasting for >30s). Clinical data were abstracted from the charts by a board-certified cardiologist.

### CMR acquisition

CMR was performed on one of the following CMR units: 1.5-T Magnetom Avanto (Siemens Healthcare, Erlangen, Germany) or Signa HDx Twin Speed (GE Healthcare, Waukesha, Wisconsin, USA) scanner or a 3.0-T Magnetom Verio scanner (Siemens Healthcare). CMR scanners were equipped with either a 32-element or an eight-element cardiac array coil. Typical steady-state free precession parameters were as follows: spatial resolution of 1.3–1.5 x 1.3–1.5 mm, section thickness of 6–10 mm and gap of 0–2 mm; and temporal resolution of 35–50 msec. LGE was performed with either segmented gradient-recalled echo or single-shot steady-state free precession inversion-recovery sequences 8–10 min after intravenous administration of 0.2 mmol/kg gadobutrol (Gadovist; Bayer Healthcare, Berlin, Germany).

### Image analysis

All studies were reviewed and analyzed by a single experienced Level III CMR reader using commercially available CMR post-processing software (cvi42, Calgary, Canada). The CMR reader was blinded to all clinical data. Maximum end-diastolic wall thickness (EDWTmax) was measured manually from cardiac short-axis (or cardiac long-axis steady state free precession sequences for the apical segments where thickened). Wall thickening was considered to be present if there was an EDWTmax of >13 mm. The population was divided into those with and without wall thickening.

Semi-automated (modified centerline technique) average wall thickness measurements and volumes for each myocardial segment and LV mass were produced by the software based on a 16-segment model [17]. Semi-automated septal to lateral wall ratios (SLR) were obtained by reviewing the average segment wall thicknesses and dividing the thickest septal segment by the thickest lateral segment.

We divided the cohort into 4 subgroups based on their morphological phenotypes; 1) those without wall thickening; 2) those with apical predominant thickening; 3) those with asymmetric septal hypertrophy (ASH) (SLR >1.3) [18] and 4) those with concentric wall thickening (SLR <1.3).

Left ventricular mass was quantified including LV papillary muscles and trabeculae and was considered elevated when above 85 g/m^2^ for males and above 81 g/m^2^ for females when indexed to body surface area (LVMI) [19]. LV papillary muscle and trabecular mass and volume were measured and recorded separately [20].

Late gadolinium enhancement (LGE) sequences acquired in conventional cardiac short-axis and 4, 3 and 2-chamber orientations as well as a stack of LGE images in a 4-chamber orientation [21] were assessed qualitatively for presence and distribution of LV scar. Patients with LV scar were separated into those with “typical Fabry scar” (predominantly basal and/or mid inferolateral midwall scar) and those with other patterns of LV scar. Mild hinge point scar was formally quantified, but not considered pathological scar [22]. Semi-automated threshold-based quantification of LV and segmental scar was performed by manually adjusting a gray-scale threshold to define areas of visually-identified LGE [23]. Scar quantification was repeated on a proportion of the study population to test intraobserver and interobserver agreement. For intraobserver agreement, scar quantification was repeated a minimum of 10 months after initial analysis.

### Statistical analysis

Continuous variables were presented as median (interquartile range). Categorical variables were analyzed with Fisher’s exact test. Group comparisons were analyzed using the Mann-Whitney *U*-test. Spearman correlation coefficient was utilized to assess correlation between continuous variables. Intraobserver and interobserver agreement was assessed by correlation coefficient.

## Results

**Fig. 1:**
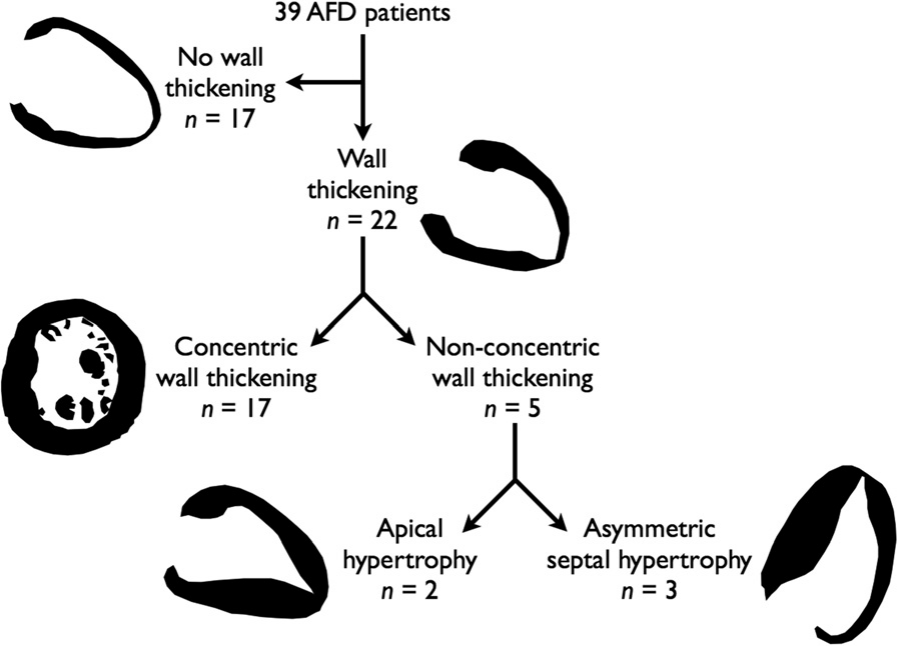
Breakdown of AFD cohort according to presence of wall thickening and the various morphological phenotypes

**Fig. 2:**
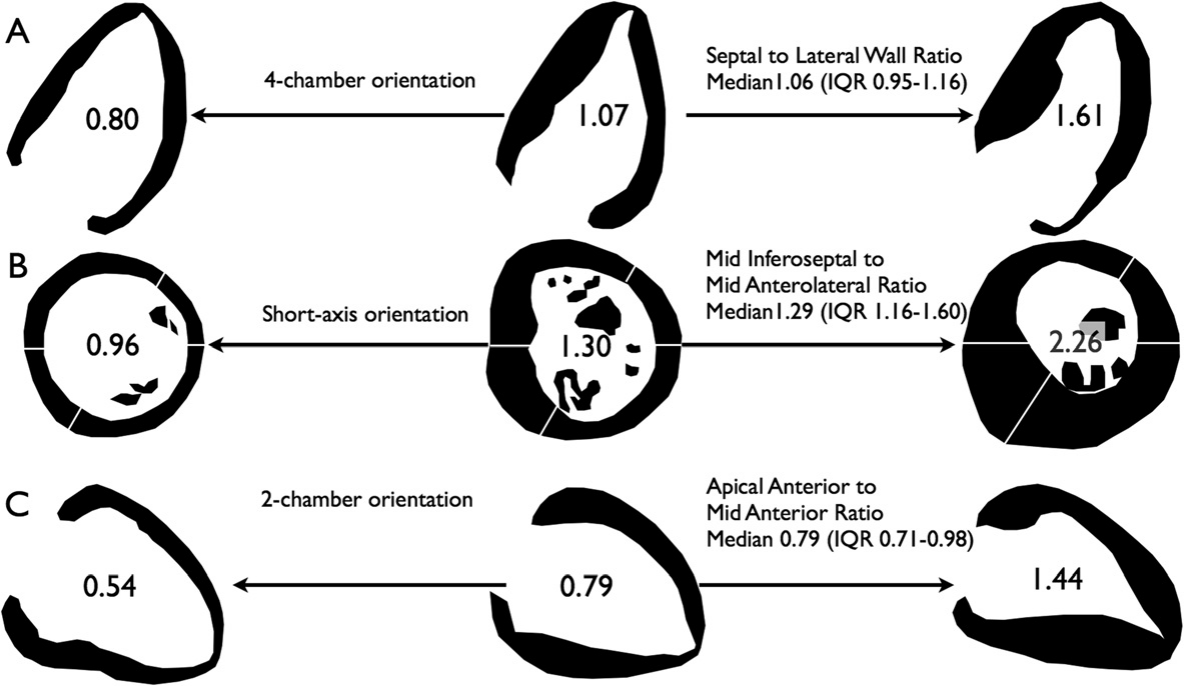
Patterns of left ventricular wall thickening in Anderson-Fabry disease (AFD). Silhouette images of 22 AFD patients with wall thickening arranged by semi-automated ratios. Cases with the median and lowest and highest values for each ratio are provided to document the full spectrum of appearances seen in AFD. The septal to lateral wall ratio (**a**) and the mid inferoseptal to mid anterolateral ratio (**b**) were chosen to highlight asymmetric septal hypertrophy and the apical anterior to mid anterior ratio was chosen to highlight preferential apical hypertrophy (**c**)

**Fig. 3:**
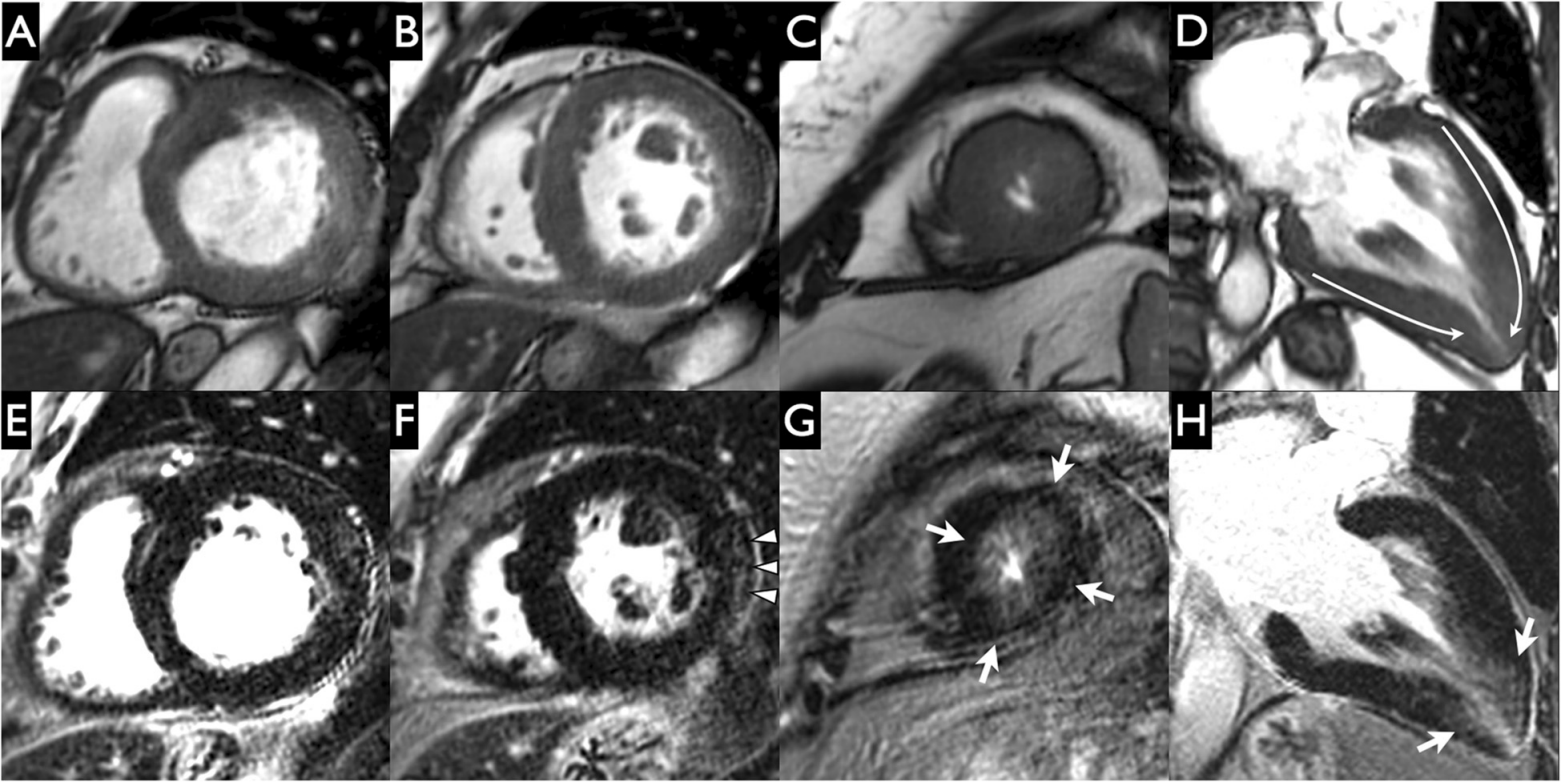
Apical hypertrophy in Anderson-Fabry Disease (AFD). Short-axis (**a**-**c**) and 2-chamber (**d**) cine steady state free precession and short-axis (**e**-**g**) and 2-chamber (**h**) late gadolinium enhancement images in a patient with Anderson-Fabry Disease on enzyme replacement therapy and a history of non-sustained ventricular tachycardia. Cardiovascular magnetic resonance revealed an apical pattern of hypertrophy (lack of apical tapering in end-diastole [white curved arrows on image D]) and obvious intermediate intensity midwall and subendocardial apical scar (white arrows on images **g**, **h**. This is not typical of ischemic heart disease - lack of high intensity myocardial scar and preserved muscle bulk. There is also subtle intermediate intensity subepicardial scar in the mid inferolateral segment (white arrowheads on image **f**. There was more scar in the apical LV than the mid and basal LV (Basal LV scar 10 %; Mid LV scar 9 %; Apical LV scar 38 %)

**Fig. 4:**
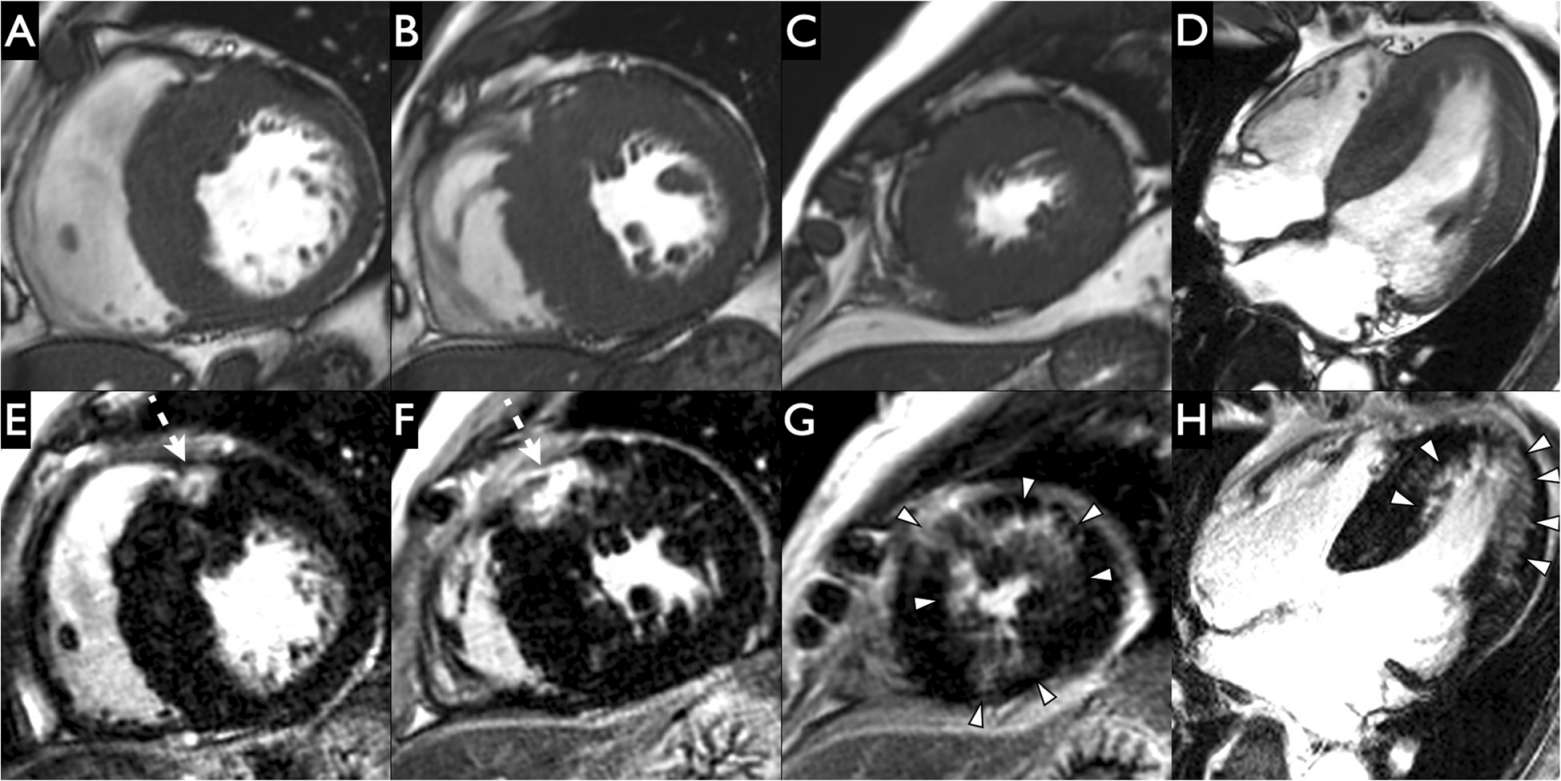
Asymmetric septal hypertrophy in Anderson-Fabry Disease (AFD). Short-axis (**a**-**c**) and 4-chamber (**d**) cine steady state free precession and short-axis (**e**-**g**) and 4-chamber (**h**) late gadolinium enhancement images in a patient with Anderson-Fabry Disease on enzyme replacement therapy. The 4 chamber view revealed a reverse septal curvature subtype of asymmetric septal hypertrophy (**d**). There is a non-ischemic pattern of scar with high intensity hinge point scar (more so in the anteroseptum than the inferoseptum - dashed arrows on images **e**-**f**. There is further intermediate intensity patchy midwall and subendocardial scar not typical of ischemic heart disease (preserved muscle bulk and patchy sparing of subendocardium and trabeculae) distributed with an apical predominance (white arrowheads on images G-H). Scar was distributed with an increasing percentage from base to apex (Basal LV scar 5 %; Mid LV scar 22 %; Apical LV scar 47 %)

**Fig. 5:**
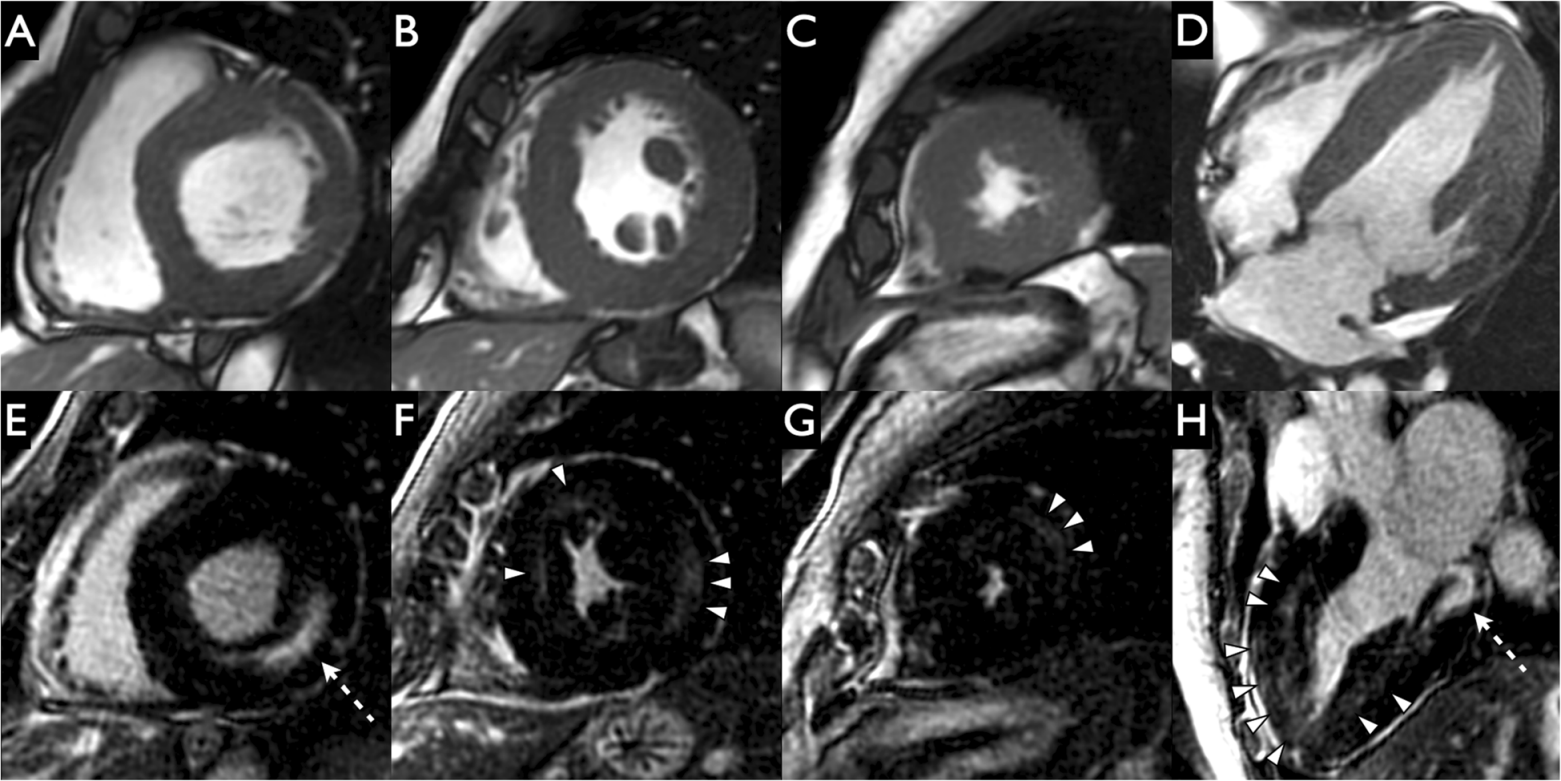
Concentric hypertrophy in Anderson-Fabry Disease (AFD). Short-axis (**a-c**) and 4-chamber (**d**) cine steady state free precession and short-axis (**e**-**g**) and 3-chamber (**h**) late gadolinium enhancement images in a patient with Anderson-Fabry disease who presented who presented initially with a wide complex tachycardia. Cardiovascular magnetic resonance imaging revealed typical concentric hypertrophy and high intensity inferolateral midwall scar (dashed white arrows in images **e** and **h**. There was also some intermediate intensity mid and apical left ventricular mid-myocardial scar (white arrowheads in images **f**-**h**. There was more scar in the basal LV than the mid and apical LV (Basal LV scar 15 %; Mid LV scar 8 %; Apical LV scar 11 %)

Thirty-nine patients met inclusion criteria (41 patients with confirmed AFD were identified from clinic database, 1 patient had CMR but no LGE sequences due to renal impairment and 1 patient was excluded due to an apical myocardial infarct. Patient characteristics are given in Table 1). Twenty-two (56 %) had wall thickening (Fig. 1). Patients with wall thickening were more likely to be male (68 % vs. 29 %, *p* = 0.025) and older (49.9 years [44.9–57.0] vs. 38.9 years [30.7–46.5], *p* = 0.008). The septal to lateral wall ratio ranged from 0.76 to 1.61 (median 1.00 [0.92–1.08], Fig. 2). Two (5 %) had an apical predominant pattern of hypertrophy (Figs. 2 and 3), 3 had ASH morphology (8 %, Fig. 4) and the remainder had concentric wall thickening (*n* = 17, 44 %, Fig. 5). For statistical analysis, the asymmetric wall thickening group and apical thickening group were collapsed into a single ‘non-concentric wall thickening’ group (*n* = 5), Fig. 1. Patients with non-concentric wall thickening had significantly greater EDWTmax than those with concentric wall thickening and there were trends towards greater LVMI (*p* = 0.066) (Figs. 3 and 4, Table 2).

**Table 1:** Clinical characteristics of patients in the cohort

Parameter	Result
Age in years (Interquartile range)	45.2 (34.7–55.5)
Males	20 (51 %)
Systemic hypertension	14 (36 %)
New York Heart Association Class I/II	29 (74 %)/10 (26 %)
Atrial fibrillation	5 (13 %)
Ventricular tachycardia	5 (13 %)
Device – Permanent pacemaker/Automated implantable cardioverter defibrillator	1 (3 %)/3 (8 %)
Enzyme replacement therapy	27 (69 %)
Renal disease	
Proteinuria	16 (41 %)
Dialysis/Kidney Transplant	3 (8 %)/3 (8 %)
Other clinical manifestations	
Stroke/Transient ischemic attack	12 (31 %)
Auditory manifestations	25 (64 %)
Acroparesthesia	26 (67 %)
Gastrointestinal tract involvement	14 (36 %)
Cutaneous manifestations	18 (46 %)

**Table 2:** Comparisons between subgroups with concentric wall thickening and non-concentric wall thickening

	Concentric wall thickening (*n* = 17)	Non-concentric wall thickening (*n* = 5)	Statistical significance (*p*-value)
Males	12 (71 %)	3 (60 %)	1.000
Age (years)	49.4 (44.1–53.9)	62.2 (51.2–63.8)	0.055
Hypertension	5 (29 %)	4 (80 %)	0.115
EDWTmax (mm)	14.4 (13.3–16.5)	17.3 (16.5–31.0)	0.017
LVEDVI (ml/m^2^)	83.6 (74.1–108.6)	78.0 (76.4–94.8)	0.845
LVEF (%)	59.3 (56.3–64.6)	57.4 (56.0–66.7)	0.969
LVMI (g/m^2^)	91.5 (77.2–103.1)	132.7 (93.1–174.7)	0.066
LVPMI (g/m^2^)	4.8 (3.6–6.4)	5.6 (4.9–9.1)	0.147
LVTPMI ml/m^2)^	13.8 (10.1–17.0)	20.2 (15.5–22.6)	0.066
Scar as percentage of total LV myocardium (%)	2.8 (1.3–7.0)	14.7 (7.1–21.8)	0.026
Scar as percentage of apical myocardium (%)	0.3 (0.0–1.1)	18.9 (14.4–40.6)	0.003
Scar as percentage of mid-ventricular myocardium (%)	2.0 (1.0–5.2)	9.1 (6.8–21.9)	0.014
Scar as percentage of basal myocardium (%)	4.4 (1.1–8.1)	5.3 (1.4–13.8)	0.411

All data are provided as numbers, percentages or interquartile range where appropriate

*EDWTmax* Maximum end-diastolic wall thickness, *LVMI* Indexed left ventricular mass (excluding papillary muscles), *LVPMI* Indexed left ventricular papillary mass, *LVTPMI* Indexed left ventricular trabecular and papillary muscle volume, *LVEDVI* Indexed left ventricular end-diastolic volume, *LVEF* Left ventricular ejection fraction

### LGE analysis

**Fig. 6:**
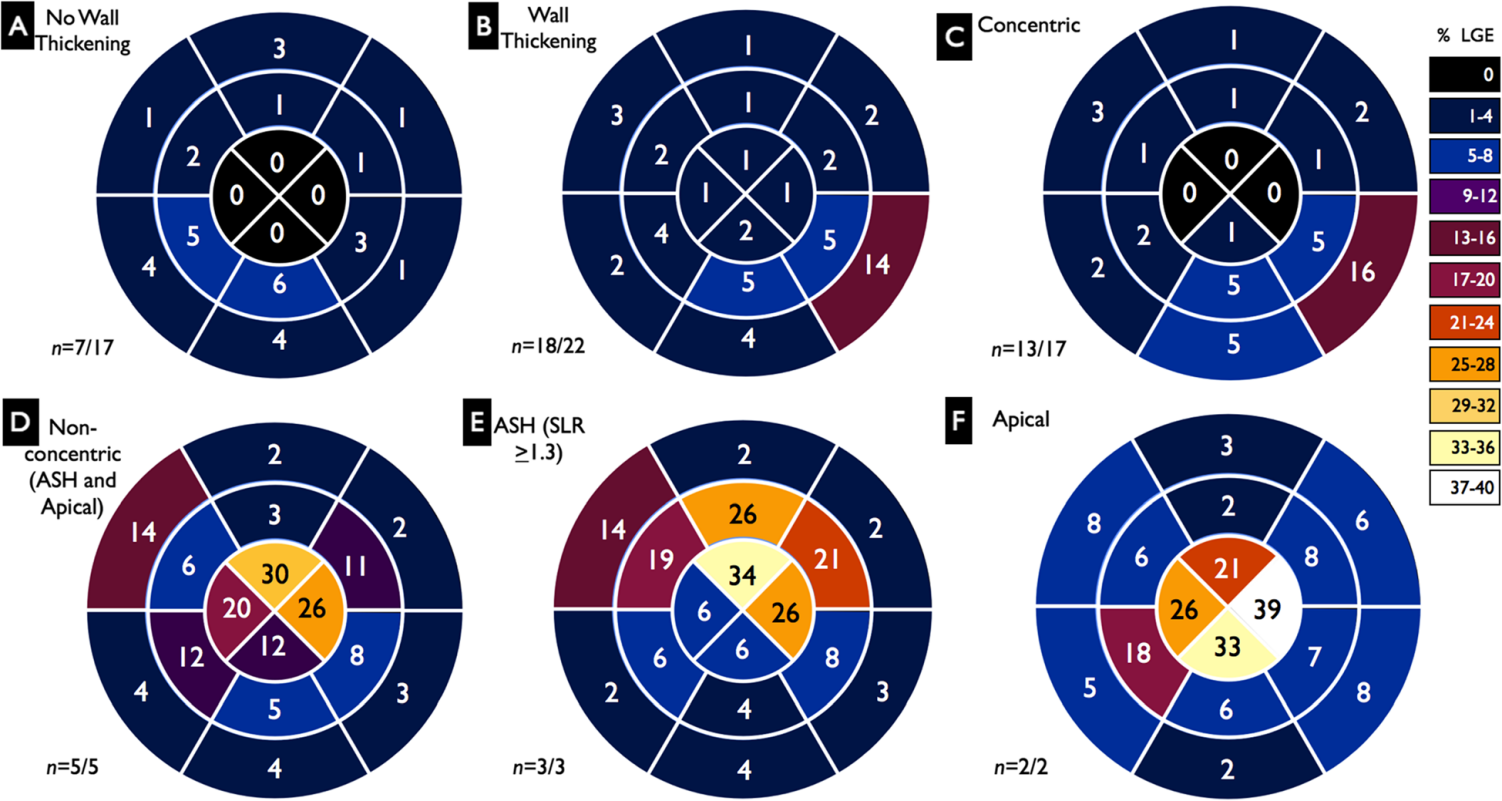
Scar patterns seen in AFD subdivided according to presence of wall thickening and morphological phenotypes. Visual demonstration of scar patterns seen in Anderson-Fabry disease subdivided according to presence of wall thickening and morphological phenotypes using color shading on a 16-myocardial-segment model of the left ventricle. Patients without late gadolinium enhancement were excluded. Numerical values were assigned to each segment equivalent to the (median) percentage of the segment that was scarred. The population was divided into subgroups with and without thickening (**a**-**b**) and again according to morphological phenotypes comparing concentric (**c**) and non-concentric (**d**), ASH (**e**), and apical hypertrophy (**f**) subgroups. ASH = Asymmetric septal hypertrophy. Non-concentric = patients with asymmetric septal hypertrophy + patients with apical predominant hypertrophy

**Fig. 7:**
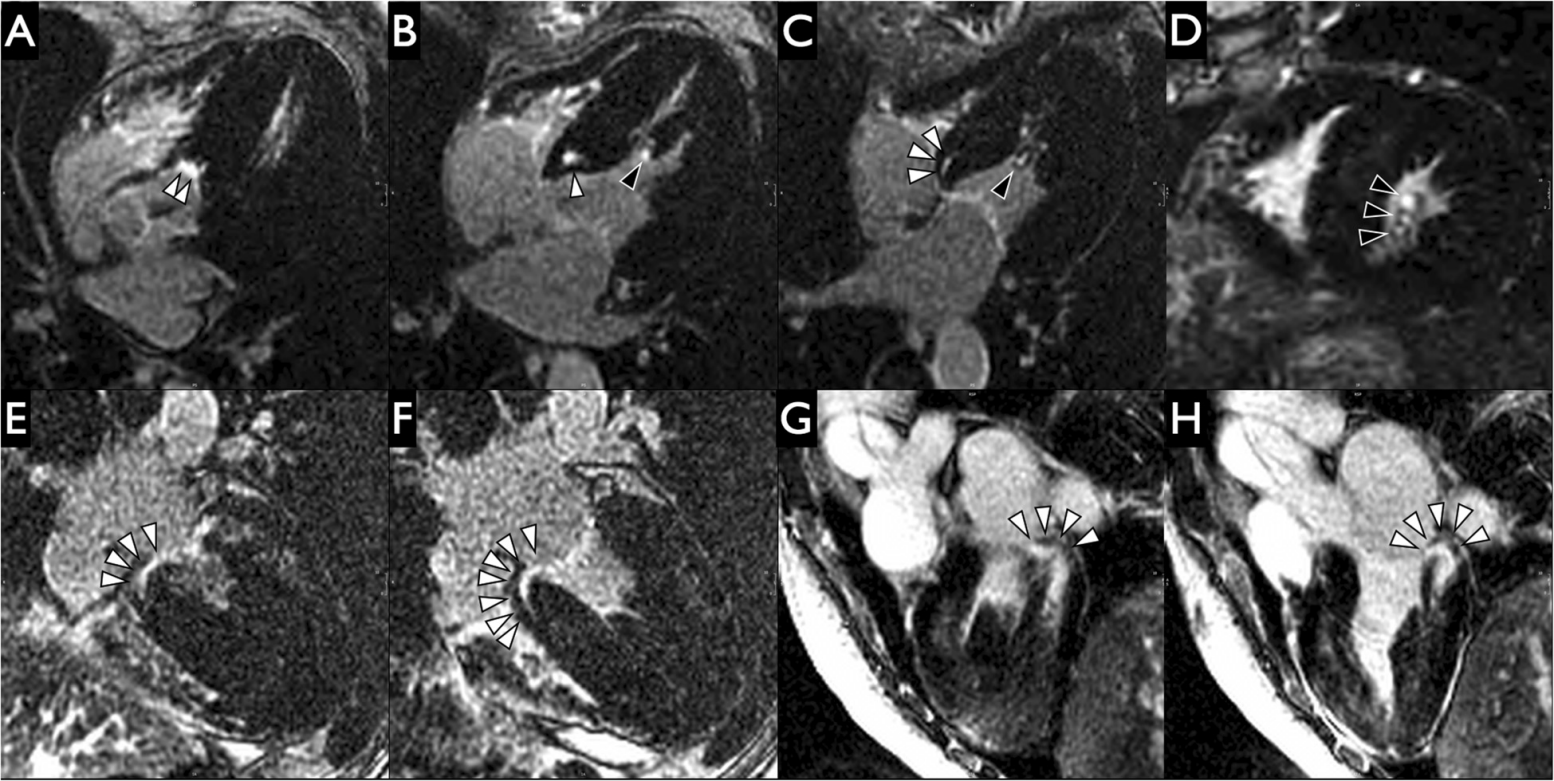
Demonstration of scar at fibrous-muscular junctions in Anderson-Fabry disease (AFD). Late gadolinium enhancement images from 2 AFD patients in our cohort. On the 4-chamber stack and cardiac short-axis late gadolinium enhancement images from a patient on ERT (**a**-**d**), high intensity midwall scar is seen to extend from the aortic annulus into the basal anteroseptal segment (white arrowheads with black border on image A) and spiral downwards into the basal inferoseptal segment (**b**-**c**). Cardiac short-axis image (**d**) and more inferior slices from the 4-chamber stack (**b-c**) demonstrate high intensity scar in the posteromedial papillary muscle group at the junction between the heads and the chordae tendinae (black arrowheads with white border). On the 4-chamber orientation stack images (**e**-**f**) from another patient on enzyme replacement therapy (ERT), there is a focus of high intensity midwall scar extending from the mitral annulus into the basal inferoseptal segment (white arrowheads with black border) in this. The 4-chamber orientation stack images (**e**-**f**) demonstrate high intensity midwall scar extending from the mitral annulus into the basal inferoseptal segment (white arrowheads with black border). 3-chamber stack images from the same patient (**g**-**h**) demonstrate high intensity midwall scar extending from the mitral annulus into the basal inferolateral segment (white arrowheads with black border)

Twenty-five patients (64 %) had evidence of LGE (Fig. 6, Additional file 1). Eight had predominantly mild hinge point fibrosis and the remaining 17 had pathological scar. Of these 17 with pathological scar, 13 (76 %) had a typical pattern of LGE. Of the 17 with pathological scar, 1 (6 %) had multifocal scar without inferolateral wall predominance, 2 (12 %) had predominantly apical scar and 1 (6 %) had mild inferior wall midwall scar. Nine of 17 (53 %) patients with concentric wall thickening had typical inferolateral scar. Of the 13 patients with typical inferolateral scar, 8 (62 %) had additional scar elsewhere in the LV, but not more than in the inferior and lateral walls. 5 of the 17 (29 %) patients with pathological scar had scar at the interfaces at fibrous-muscular junctions (at insertion points of the valve leaflets into the myocardium and at the interface between the chordae tendinae and papillary muscles). These were better demonstrated on stacks of 4-chamber orientation LGE images (Fig. 7).

There was moderate correlation between total LV scar and EDWTmax (rho = 0.52, *p* = 0.001) as well as between total LV scar and LVMI (rho = 0.44, *p* = 0.001. Patients with non-concentric wall thickening (*n* = 5) had more total LV scar, apical LV scar and mid ventricular LV scar than patients with concentric wall thickening (*n* = 17) (Table 2, Fig. 6).

Interobserver agreement was assessed utilizing the 17 cases with pathological scar. Concordance correlation coefficient was 0.93 for intraobserver agreement (95 % CI, 0.83 to 0.97) with minimal bias (mean -2.6 g, *p* = 0.141) and, 0.98 for interobserver agreement (95 % CI, 0.92 to 0.99) with minimal bias (mean -0.6 g, *p* = 0.528).

### Correlation between imaging and clinical parameters

Twenty-four patients (62 %) had both extracardiac and cardiac involvement (‘classic variant’); 11 patients (28 %) had only extracardiac involvement; and 4 patients (10 %) were carriers. There were no patients in our cohort with only cardiac manifestations of AFD (‘cardiac variant’). The majority of ‘classic variant’ patients had wall thickening (92 %), pathological LGE (71 %), typical inferolateral scar (54 %), and concentric wall thickening (71 %). All patients with non-concentric wall thickening had ‘classic variant’ AFD.

There was more arrhythmia in patients with elevated LVMI (8/16 with elevated LVMI vs. 2/23 with normal LVMI, *p* = 0.007). Patients with arrhythmia (*n* = 10) had greater LVMI than those without arrhythmia (*n* = 31), (111.8 g/m^2^ [103.0–165.0] vs. 75.9 g/m^2^ [66.0–89.5], *p* = 0.003). Patients with ventricular arrhythmia (*n* = 5) had significantly greater LVMI and LV papillary muscle mass (Table 3). Patients with sustained ventricular tachycardia (*n* = 2) had greater LVMI, papillary mass and trabecular and papillary muscle volume (all *p* = 0.02). Elevated LVMI was associated with ventricular arrhythmia (4/9 patients with LVMI >125 % of the upper limit of normal vs. 1/30 with LVMI below this level *p* = 0.009) and sustained ventricular tachycardia (2/5 patients with LVMI >150 % of the upper limit of normal vs. 0/34 with LVMI below this level *p* = 0.013,and 2/4 patients with LVMI >175 % of the upper limit of normal vs. 0/35 with LVMI below this level *p* = 0.008) respectively. There was a significant association between elevated trabecular and papillary muscle volume and all arrhythmia (*p* < 0.001), ventricular arrhythmia (*p* = 0.010) and sustained ventricular tachycardia (*p* = 0.013). There was no significant association between LA volume and atrial fibrillation.

**Table 3:** Comparison between patients with and without ventricular arrhythmia

	All ventricular arrhythmia (*n* = 5)	No ventricular arrhythmia (*n* = 34)	Statistical significance (*p*-value)
Total LV mass (g/m2)	132.7 (96.8–193.2)	78.5 (66.1–94.8)	0.029
LV papillary muscle mass (g/m2)	8.6 (5.8–11.0)	4.0 (3.5–5.4)	0.036
Trabecular and papillary muscle volume (ml/m2)	20.2 (12.2–28.1)	11.9 (9.7–16.6)	0.065
Total LV scar (%)	8.9 (0.0–12.3)	1.7 (0.0–4.1)	0.444
Total apical scar (%)	10.6 (0.0–23.8)	0.1 (0.0–0.9)	0.299
Total mid-ventricular scar (%)	8.3 (0.0–8.6)	1.8 (0.0–4.9)	0.495
Total basal scar (%)	1.3 (0.0–11.3)	2.2 (0.0–7.2)	0.966

*LV* left ventricular

## Discussion

Previous CMR literature on AFD has focused on scar patterns in AFD. Although CMR, with its high spatial resolution, is ideally suited to assess patterns of LV wall thickening [24], to our knowledge, this is the first paper in the CMR literature that has set out to document the spectrum of wall thickening patterns in AFD and by implication, the potential overlap with HCM with which it may be confused. The echocardiographic literature on this subject (summarized in Additional file 2) confirms that AFD can manifest as ASH and apical hypertrophic patterns of wall thickening with variable proportions. In our cohort, we were able to identify subgroups of patients with concentric wall thickening (19/24, 79 %) and non-concentric wall thickening (5/24, 21 %). The non-concentric wall thickening subgroup included patients with asymmetric septal and apical hypertrophic patterns and we found that these patients had greater wall thicknesses and LVMI than patients with concentric wall thickening. In one previously reported cohort, over two thirds of previously undiagnosed AFD patients (ranging in age between 47 and 79 years) had an ASH pattern of wall thickening [7], which is at odds with our own cohort in whom only one quarter demonstrated asymmetric thickening.

Published CMR findings to date have emphasized the typical pattern of myocardial scar seen in AFD [14, 16, 25–27] (Additional file 1). However, it was our objective to obtain a clear picture of the spectrum of disease expression in this rare condition and we found substantial variability in scar burden and distribution. We found that four fifths (79 %) of patients with pathologic LGE had typical inferolateral scar but that almost two thirds (60 %) of those with inferolateral scar had other LV scar as well. Therefore, in our cohort, a fifth (21 %) of patients with pathological LGE had atypical patterns of scar. Furthermore, patients with non-concentric thickening had more LV scar and more mid and apical LV scar than those with concentric thickening. Previous echocardiography-based research into a small cohort of asymmetric septal hypertrophy patterns in AFD revealed more short term adverse events, which was statistically linked to increased asymmetry and ‘thinning’ of the posterior wall, but there was no myocardial characterization [7]. We found a higher percentage of patients (24 %) with atypical scar distribution than most of the reported cohorts (Additional file 1), emphasizing the importance of including AFD in the differential diagnosis of hypertrophic myocardial disease. Although a recent study has described LGE in myocardial segments outside the typical locations, further detail or correlation with other imaging or clinical parameters was not provided [15]. Our data suggest that patients with atypical patterns of wall thickening have more total scar as well as more apical and mid ventricular scar, whereas most AFD patients have basal inferolateral LGE. Recent studies have identified non-contrast T1 mapping as a highly sensitive and specific early marker of cardiac involvement in AFD [28–30]. At present, however, this sequence remains in the research arena and was not utilized in evaluation of our retrospective cohort. The presence of LGE remains an important biomarker of disease since it is one of the indicators that permits funded treatment with enzyme replacement therapy.

In addition, we found 5 patients with midwall scar at fibrous-muscular interfaces including valve annuli/LV wall and chordae tendinae/papillary muscle heads respectively, which to our knowledge has not been described in the imaging literature to date. The presence of papillary scar is unsurprising given the prior reports of enlarged papillary muscles and disproportionate contribution of trabeculae and papillary muscles to total LV mass in AFD [15, 31]. We hypothesize these interfaces between the fibrous skeleton of the heart and the LV midwall or mesocardial layer are sites of increased stress. Viewing the mitral annulus as a bucket handle and considering the basal anteroseptum as the least mobile basal segment similar to the hinge of the handle, the basal inferolateral segment (diagonally opposite on the clock face) is the most mobile of the basal segments and likely faces the most junctional stresses transmitted from the fibrous skeleton into the mesocardial layer. Images G-H from Fig. 7 demonstrate continuity of the scar from the mitral annulus into the mesocardial layer/midwall of the basal inferolateral segment. This hypothesis may explain why midwall basal inferolateral scar is the most common pattern of myocardial scar in AFD.

Our analysis was limited by the small sample size, but this is to be expected given of the rarity of the disease, estimated at between 1 in 7000–8000 live births [32]. For the first time in the literature, our findings have linked the CMR phenotype to arrhythmia. Intriguingly, our data raise the possibility of a link between elevated LV mass and ventricular arrhythmia, as is also seen in HCM in which significant hypertrophy predicts adverse events [33]. We found that patients with elevated LV mass had more overall arrhythmia and that greater degrees of LV hypertrophy, were associated with ventricular arrhythmia and sustained ventricular tachycardia. Increased trabecular and papillary muscle volume above the normal range was associated with overall arrhythmia, atrial fibrillation, ventricular arrhythmia and sustained ventricular tachycardia. Given the disproportionate contribution that LV papillary muscle and trabeculations make to LV mass [15], this finding may be a surrogate marker for disease severity in Fabry disease rather than the actual cause of any arrhythmia.

While we found that patients with both ventricular arrhythmia of any duration had more LV scar, our sample size was too small to prove a definite association. Lastly, multivariable logistic regression was not possible due to the small sample size. As non-concentric patterns of wall thickening were associated with increased LV mass and scar, larger multicenter long term outcome studies focusing on AFD patients with these patterns of hypertrophy and apical predominant scar may be helpful in teasing out other risk factors for ventricular arrhythmia.

## Conclusions

Our cohort demonstrates that Anderson-Fabry disease has a number of different phenotypic expressions both in extent and location of hypertrophy and pattern of scar. There is consequently a direct overlap between the AFD phenotype and HCM phenotype. It is therefore unsafe to rely on imaging appearances alone when trying to exclude or make the diagnosis of AFD, and genetic testing is still indicated where there is still a reasonable degree of clinical suspicion. Some centers, including ours, offer AFD screening to all potential HCM patients because of the existence of AFD variants with only cardiac manifestations. Concentric thickening and inferolateral mid-myocardial scar are the most common manifestations of AFD, but the spectrum includes cases morphologically identical to apical and ASH subtypes of HCM and these have more apical and mid-ventricular LV scar than cases with concentric thickening. Further research into differences in clinical outcomes between concentric and non-concentric morphologic subtypes is warranted in larger cohorts. Patients with elevated indexed left ventricular mass in our cohort had a greater incidence of ventricular arrhythmia. Further research collaborations should focus on the potential link between LV mass, LV scar burden and distribution, and ventricular arrhythmia in Anderson Fabry Disease.

## Additional files

Additional file 1: Patterns of late gadolinium enhancement in the current study population compared to existing literature. (DOC 37 kb) Additional file 2: Patterns of hypertrophy in the current study population compared to existing literature. (DOC 49 kb)

## Abbreviations

AFD: Anderson-Fabry disease
ASH: Asymmetric septal hypertrophy
CI: Confidence interval
CMR: Cardiovascular magnetic resonance
EDWTmax: Maximum end-diastolic wall thickness
eGFR: Estimated glomerular filtration rate
LGE: Late gadolinium enhancement
LVMH: Left ventricular mass in grams indexed to height in meters
LVMI: Left ventricular mass in grams indexed to body surface area
SLR: Semiautomated septal to lateral wall ratio
