# Supplementary material for: Cardiovascular magnetic resonance demonstration of the spectrum of morphological phenotypes and patterns of myocardial scarring in Anderson-Fabry disease
Source: J Cardiovasc Magn Reson. 2016 Mar 31;18:14. doi: 10.1186/s12968-016-0233-6 (PMC4818406; doi:10.1186/s12968-016-0233-6)
Supplement: Additional file 1: — Patterns of late gadolinium enhancement in the current study population compared to existing literature. (DOC 37 kb) [file 12968_2016_233_MOESM1_ESM.doc]

**Additional file 1 Patterns of late gadolinium enhancement in the current study population compared to existing literature**

| First Author, *Journal,* Year of Publication | Patients with AFD | Late gadolinium enhancement (LGE) | Classic pattern (basal inferolateral sparing the subendocardium) | Other patterns | LVH patterns |
| --- | --- | --- | --- | --- | --- |
| Current study population | 39 | 17 patients had pathological LGE* (44%). 13 of 17 (76%) had typical predominantly inferolateral LGE.  *8 additional patients (21%) had mild hinge point LGE. | 13 of 17 (76%) | 1 of 17 (6%) had multifocal LGE without inferolateral predominance, 2 of 17 (12%) had apical predominant LGE, 1 (6%) had mild inferior wall midwall LGE. | See other tables |
| Moon, *Eur Heart J,* 2003 | 26 | 13 (50%). 12 of 13 patients had basal inferolateral LGE. | 12 of 13 (92%) | 1 of 13 patients (8%) had inferior LGE but not basal lateral wall LGE. | N/A |
| Beer, *Am J Cardiol,* 2006 | 35 | 11 (31%). 8 of 17 scheduled for ERT had LGE. 6 of 8 had inferior or lateral wall LGE. | 6 of 8 (75%) of those scheduled for ERT | 2 of 8 (25%) anterior or septal walls. | N/A |
| Pieroni, *J Am Coll Cardiol,* 2006 | 40 | 10/40 (25%). 10 of 10 had basal or basal to mid lateral or inferolateral segment LGE. | 10 of 10 (100%) | 1 of 10 patients (10%) had additional focal apical midwall LGE (had more severe LVH) | N/A |
| De Cobelli, *Am J Roentgenol,* 2009 | 13 | 10 (77%). 10 of 10 had inferolateral LGE. | 10 of 10 (100%) | 2 of 12 had additional anteroseptal LGE (17%). | All concentric |
| Niemann, *JACC: Cardiovasc Imaging,* 2011 | 104 | 41 (39%). 41 of 41 had basal inferolateral LGE. | 41 of 41 (100%) | Additional LGE seen in these patients in basal inferoseptal (*n*=2, 5%), basal anteroseptal (*n*=8, 20%) and 1 mid inferoseptal segments (*n*=1, 2%) | N/A |
| Sado *Circ: Cardiovasc Imaging* 2013 | 44 | Not disclosed. 6 patients with AFD did not receive gadolinium (4 due to chronic renal failure, 2 due to patient preference). | Patterns of LGE were not disclosed in the 20 patients with LVH who received contrast. 4 of 18 patients without LVH who received contrast had LGE in the basal inferolateral wall. | N/A | N/A |
| Thompson *Circ: Cardiovasc Imaging* 2013 | 31 | 13 of 29 (45%) patients had positive LGE studies | The basal lateral wall was the most common location for LGE | N/A | N/A |
| Kozor *Heart* 2016 | 50 | 6 patients with AFD did not receive gadolinium due to severe renal impairment. 15 of the 44 (34.1%) had LGE. | 14 of 15 (93%) | Additional LGE seen in the basal inferoseptal (15%) and basal anterior (7%) and mid anterior segments (7%) | N/A |

AFD – Anderson-Fabry disease; eGFR – estimated glomerular filtration rate
