# Supplementary material for: Cardiovascular magnetic resonance demonstration of the spectrum of morphological phenotypes and patterns of myocardial scarring in Anderson-Fabry disease
Source: J Cardiovasc Magn Reson. 2016 Mar 31;18:14. doi: 10.1186/s12968-016-0233-6 (PMC4818406; doi:10.1186/s12968-016-0233-6)
Supplement: Additional file 2: — Patterns of hypertrophy in the current study population compared to existing literature. (DOC 49 kb) [file 12968_2016_233_MOESM2_ESM.doc]

**Additional file 2. Patterns of hypertrophy in the current study population compared to existing literature**

| First Author, *Journal,* Year of Publication | Patients with AFD | Wall thickening (as a percentage of cohort) | Without wall thickening (as a percentage of cohort) | Concentric hypertrophy (as a percentage of those with wall thickening) | Asymmetric septal hypertrophy  (as a percentage of those with wall thickening) | Other patterns (as a percentage of those with wall thickening) |
| --- | --- | --- | --- | --- | --- | --- |
| Current study population | 39 | 22 (56%) | 17 (44%) | 17 (77%) | 3 (14%) | 2 with apical hypertrophy (9%) |
| Bass *Am Heart J* 1980 | 32 | 15 (48%) | 17 (52) | 13 (87%) | 2* (13%) | 0 (0%) |
| Nakao *N Eng J Med* 1995 | 7 | 7 (100%) | 0 (0%) | 7 (100) | 0 (0%) | 0 (0%) |
| Linhart *Am Heart J* 2000 | 30 | 14 (47%) | 16 (53%) | 11 (79%) | 3 (21%) | 0 (0%) |
| Sachdev *Circulation* 2002 | 6 | 6 (100%) | 0 (0%) | 5 (83%) | 1 (17%) | 0 (0%) |
| Chimenti *Circulation* 2004 | 4 | 4 (100%) | 0 (0%) | 2 (50%) | 1 (25%) | 1 with apical hypertrophy (25%) |
| Kawano *Am J Cardiol* 2007 | 13 | 13 (100%) | 0 (0%) | 4 (31%) | 9 (69%) | 0 (0%) |
| Wu *European Heart Journal* 2010 | 139 | 118 had increased LVMI (85%) | 21 had normal LVMI (15%) | 115 (97%) | 3 (3%) | 0 (0%) |
| Elliott *Heart* 2011 | 7 | 7 (100%) | 0 (0%) | 4 (57%) | 3 (43%) | 0 (0%) |
| Sado *Circ: Cardiovasc Imaging* 2013 | 44 | 24 had increased LVMI (55%) | 20 (45%) had normal LVMI | N/A | N/A | N/A |
| Thompson *Circ: Cardiovasc Imaging* 2013 | 31 | 16 had increased LVMI (52%) | 15 had normal LVMI (48%) | N/A | N/A | N/A |
| Kozor *Heart* 2016 | 50 | 19 had increased LVMI (38%) | 31 had normal LVMI (62%) | N/A | N/A | N/A |

LVMI – left ventricular mass index. *9 patients had a septal to lateral wall ratio of 1.3 or greater, but only 2 of these had a maximum septal wall thickness of 13mm or greater.
